# A general theory of multistable systems in pathophysiology

**DOI:** 10.12688/f1000research.123183.1

**Published:** 2022-08-05

**Authors:** Bruno Burlando

**Affiliations:** 1 Department of Pharmacy, University of Genoa, Genoa, 16132, Italy

**Keywords:** bistable switch, feedback loops, pathogenesis, pathophysiology, systems and control theory, systems biology

## Abstract

Despite intensive investigations numerous diseases remain etiologically puzzling and recalcitrant to treatments. A theory is proposed here assuming that these difficulties are due to an unsuitable approach to the mechanisms of life, which is subjugated by an apparent complexity and fails to grasp the uniformity that lays behind. The stability of metabolism, despite the enormous complex of chemical reactions, suggests that reciprocal control is a prerequisite of life. Negative feedback loops have been known for a long time to maintain homeostasis, while more recently, different life processes involved in transitions or changes have been modeled by positive loops giving rise to bistable switches, also including various diseases. The present theory makes a generalization, by assuming that any functional element of a biological system is involved in a positive or a negative feedback loop. Consequently, the theory holds that the starting mechanism of any disease that affects a healthy human can be conceptually reduced to a bistable or multistationary loop system, thus providing a unifying model leading to the discovery of critical therapeutic targets.

## Introduction

Complexity is one of the most addressed features of life and a possible major hindrance to the advancement of knowledge in life science. This inevitably makes life science a non-exact scientific field largely operating through qualitative, verbose descriptions, as opposed to the quantitative, mathematical models of physics. Consequently, the predictivity of theoretical models in life science is generally low, posing serious limits to their application to the real world. This is mirrored by serious difficulties in biomedical investigations, with several diseases remaining etiologically puzzling and recalcitrant to treatments, including tumor malignancies, neurodegenerative diseases, immune disorders, metabolic syndromes, and different infectious diseases. However, rather than being due to the excessive complexity of life, these drawbacks could derive from an unsuitable approach to the study of life processes, including transitions from physiological to pathological conditions.

The human body is believed to consist of about 10 ^13^ cells ( Bianconi *et al*., 2013), while about 10 ^10^ s ^-1^ chemical reactions can be roughly calculated to occur inside each cell, considering basal metabolism and the energy released per mole by ATP hydrolysis ( Wackerhage *et al*., 1998), thus making up a total of about 10 ^23^ s ^-1^ chemical reactions in the whole body. The ability of maintaining a metabolic steady state, despite such a huge complex of events, seems statistically paradoxical. However, this would be true if metabolic processes were uncorrelated from each other, whereas they are supposed to be strictly regulated and cross-adjusted ( Burlando, 2017; El-Samad, 2021). Hence, if reciprocal control is the prerequisite of life processes, their study cannot be exempted from an appropriate analysis of control mechanisms.

Seminal theoretical work by Ludwig Von Bertalanffy has led to the development of systems theory ( von Bertalanffy, 1968), which in the beginning was inspired by living systems, and thereafter was widely applied to engineering. Thereafter, systems and control theory has developed the study of dynamic systems consisting of negative or positive feedback loops, i.e. sequences of interactions among functional agents arranged as closed chains ( Blanchini *et al*., 2014). Negative loops have an uneven number of inhibitory steps, in addition to possible activation steps, and one stable equilibrium point or a limit cycle with oscillatory behavior. By contrast, positive loops have an even number of inhibitory steps, or none, and admit multiple stable equilibrium points.

Physiological mechanisms based on negative feedback loops are known to maintain homeostasis, or induce oscillations within fixed boundaries, thus ensuring the body’s steady state ( Blanchini *et al*., 2014; Burlando *et al*., 2019). Conversely, positive feedback loops have been classically underestimated and assumed to occur sparingly, due to their supposed destabilizing effect in need of compensation by negative loops. However, the advent of systems biology has favored a new analysis of life complexity, by focusing not so much on the biodiversity of life constituents, but rather on the interactions that are established between them ( Wolkenhauer and Mesarovic, 2005; Wolkenhauer *et al*., 2005). Such a new trend has proved that several life processes, especially those that produce irreversible changes, can be modeled by positive loops. A noncomprehensive list includes bistable gene expression ( Olivenza *et al*., 2019), cell cycle ( Mochida *et al*., 2016; Vigneron *et al*., 2018; Stallaert *et al*., 2019), mitosis ( Hutter *et al*., 2017), cell migration ( Nguyen *et al*., 2018), cell differentiation ( Wang *et al*., 2009), and axon growth ( Padmanabhan and Goodhill, 2018).

## A loopomic theory in pathophysiology

The accumulating evidence that loops play an essential role in several functions accomplished by living beings ( El-Samad, 2021) has inspired the loopomics paradigm, i.e. the assumption that any functional element of a biological system is somehow involved in a loop mechanism, while the whole system can be conceptualized as an intertwined array of loops ( Burlando, 2017). Hence, the dynamics of the whole system obey to a restricted number of rules, but nevertheless, they give rise to a highly complex biodiversity, expressed in terms of epiphenomena occurring at different dimensional scales, e.g. subcellular organelles, cells, tissues, human beings, etc. Hence, given the above dynamical behavior of functional loops, any change or transition that can be observed within the human body would be the result of a multistable positive loop system switching among different equilibrium points ( Laurent and Kellershohn, 1999).

This is a completely new redefinition of life, shifting from complexity to uniformity through a comprehensive and unifying modeling in terms of loops. Such a new paradigm has deep repercussions in the field of medicine because any disease that affects a healthy organism must have at its origin an early event in the form of a change that drives the system from a physiological to an altered functional regimen. Therefore, according to the present theory this change must be driven by a positive loop.

A rapidly increasing number of pathogenic processes are being modeled as bistable switches, a few representative examples of which include cancer ( Kim *et al*., 2007; Huang *et al*., 2014), prion infections ( Kellershohn and Laurent, 2001), immunological disorders ( Beutler, 2009), dermatitis ( Dominguez-Huttinger *et al*., 2017), neurological problems ( Mucci *et al*., 2020), and neurodegenerative diseases ( De Caluwe and Dupont, 2013; Burlando *et al*., 2020). In other cases, a bistable model has not been explicitly used to describe pathogenesis, but a positive loop has been nevertheless invoked ( Beutler, 2009; Norwitz *et al*., 2019). However, this kind of approach is generally conducted on a case-by-case basis, without making any attempt at proving generalizability, despite long established evidence that could be used to move towards this direction, as explained below. First, it is obvious that diseases affecting a healthy human must involve at their origin pathophysiological changes leading to transitions from a physiological to pathological condition. Second, diseases are classifiable (e.g. https://www.who.int/standards/classifications/classification-of-diseases), i.e. they make up an ordered set, showing that the above changes are predictable. Third, diseases are recurrent through human generations, showing that pathophysiological changes depend on the activity of clonable physiological pathways that have been selected for by natural selection. Therefore, these changes must admit stability as a starting point, otherwise their selection would not have been possible. However, stability must also characterize their ending point, otherwise disease classification would not be possible. In summary, diseases depend on predictable changes carried out by multistable systems, and therefore a generalization of these processes can be achieved by using theoretical models able to develop transitions between different stable equilibrium points.

**Figure 1.**
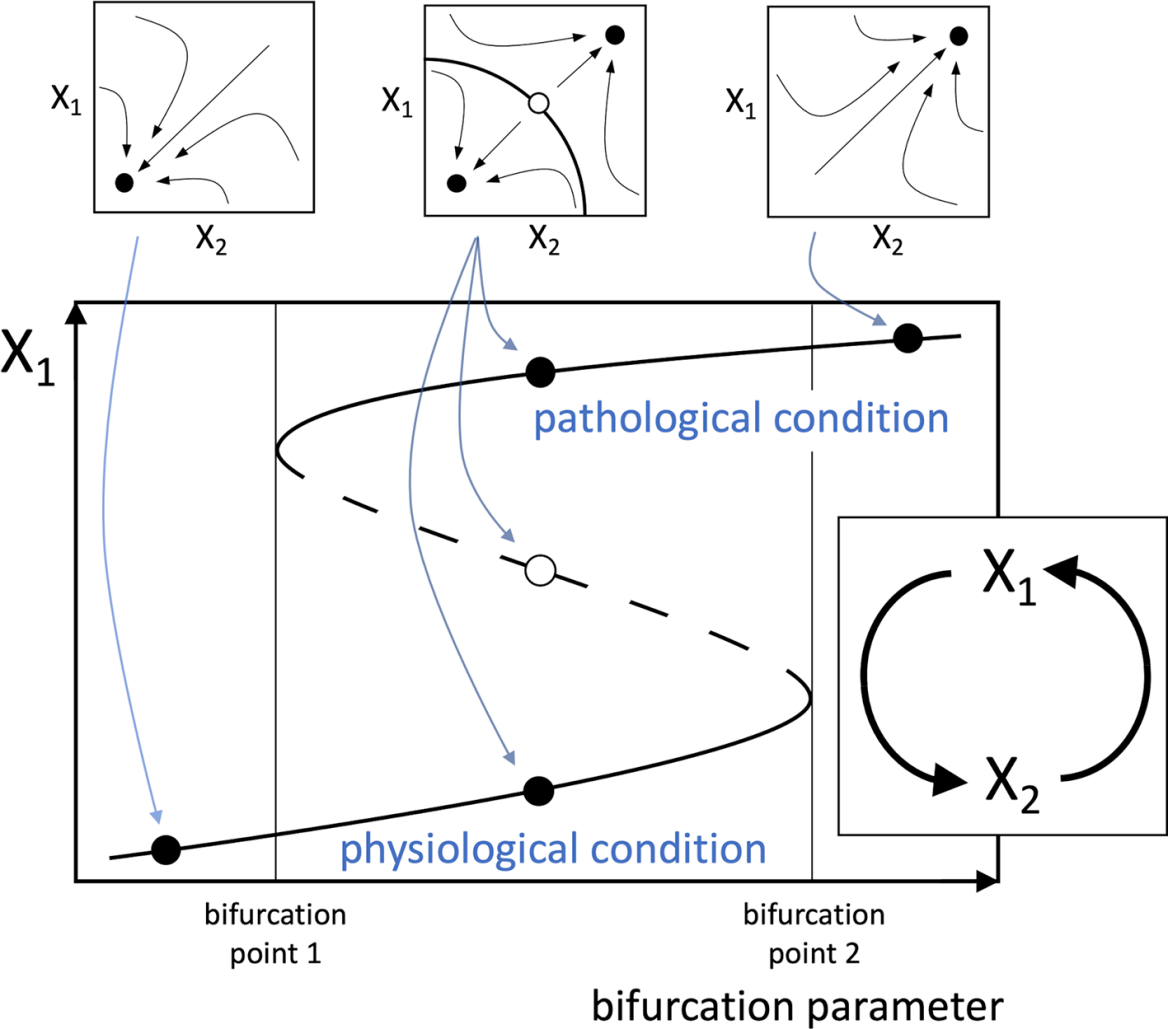
Schematic positive loop, representing a simplified model of a hypothetical pathogenic process. The illustrated system captures the essential elements of the proposed theory, though more complex loops are likely to occur in real pathophysiological processes. The loop (inset below) consists of two functional agents, indicated by X _1_ and X _2_, which may represent the amounts or activities of cells (e.g. lymphocytes), enzymes (e.g. kinases), signal molecules (e.g. interleukins), etc. In the mathematical analysis of the loop, the dynamical interactions between the functional agents are described by a system of differential equations yielding the rate of change of the functional agents. In commonly used models of biological systems dynamics, each functional agent X _i_ is assumed to undergo spontaneous inactivation and evolve along time with time constant $\tau_{X_{i}}$ . The system of differential equations, in a schematic form, is the following:

A new theory can therefore be proposed, stating that any disease that affects a healthy human involves at its primary causal event a change that can be conceptually reduced to a multistable system (typically a bistable one) depending on a positive functional loop consisting of cellular, molecular, or biochemical elements interplaying with each other. Also, the mathematical study of loop dynamics can allow the identification of bifurcation parameters that drive the transition from monostability to multistability, thus allowing the switching of the system from a “physiological” to a “pathological” stable equilibrium point, or steady state. Hence, the physical correspondents of these parameters, which could be single factors or pathways, are to be recognized as best therapeutic targets, potentially allowing the achievement of disease manageability via pharmacological or non-pharmacological treatments. Moreover, this kind of system is characterized by hysteresis, i.e. the transitions from physiological to pathological steady states occur at different threshold values of the bifurcation parameter, according to the direction of movement along the steady state trajectory, and therefore, irreversibility is also possible for a strengthening of the interactions among the functional agents of the loop ( Figure 1).

$$\tau_{X_{1}}{\dot{X}}_{1}+X_{1}=f\left(X_{2}\right)\;$$ $$\tau_{X_{2}}{\dot{X}}_{2}+X_{2}=f\left(X_{1}\right)\;$$ A possible example for the functions appearing in the differential equations is the Hill function $f\left(x\right)=\frac{\alpha x^{p}}{1+\beta x^{p}}$ , which is a suitable model for different stimulus-response relationships in biological systems ( Ferrante *et al*., 2009; Huang *et al*., 2014). However, *f*(X _1_) and *f*(X _2_) need not have the same form. By solving the system of differential equations, the trajectories followed by the system in the X _1_/X _2_ phase portrait can be traced (small plots at the top), showing the attraction basins of the stable equilibrium points (full dots), i.e. the system steady-states, and an unstable equilibrium point (empty dot). The bifurcation diagram (large plot below) shows the plot of the equilibrium values of X _1_ as a function of a bifurcation parameter, represented by one of the parameters of the differential equations. A similar plot can be derived for X _2_. The continuous lines represent stable equilibrium points and the dashed line unstable equilibrium points. As the bifurcation parameter increases (or decreases, depending on cases), the system can change from monostable (left), i.e. having a single stable equilibrium point, to bistable (middle), i.e. with two stable equilibrium points and an intermediate unstable equilibrium point, and eventually to monostable again (right). The two alternative stable equilibrium points represent the physiological and pathogenic conditions. Therefore, depending on the direction of variation, the bifurcation factor creates the conditions for the switch from physiological to pathogenic condition, or promotes the backward transition, respectively. The system also shows hysteresis, i.e. as shown in Figure 1, as the bifurcation parameter increases, and in the absence of other stimuli, the system is forced to abruptly jump from the physiological to the pathological condition at bifurcation point 2. However, once the system resides on the pathological condition, the reverse transition to physiological condition requires that the bifurcation parameter decreases until it reaches bifurcation point 1.

In operational terms, if a completely new disease is discovered, what is the main question to be addressed in order to manage it? Of course, this question concerns etiology, i.e. identifying the pathophysiological process leading to the primary causes of the disorder. Until we have an answer to this question, few possibilities exist to find a complete solution to the disease, and this is the main drawback that frequently affects biomedical research. According to the herein proposed theory, the above unexplored disease should be investigated by using currently available and newly acquired knowledge through the following steps from bench to bed: (i) localize the site of insurgence, (ii) identify major biological (molecular) factors involved, (iii) identify a positive loop (or a loop system collectively behaving as a positive loop) that could allegedly drive the pathogenic transition, (iv) develop formal mathematical analysis of the loop leading to the identification of critical bifurcation parameter(s), (v) design *in vitro* and/or *in vivo* experiments to verify that the loop and its bifurcation parameter(s) are responsible for the development of the disease. If successfully accomplished, these investigations would open the way to finely oriented pharmacological and pharmaceutical research directed to bifurcation parameter(s), followed by clinical trials.

The described approach is assumed to be the most suitable to fit the nature of life processes and therefore, given that health problems also derive from life processes, it is expected to maximize the chance of finding solutions to diseases. Although not intentionally hinged in the present theory, a study in this direction has already been done through a large-scale characterization of bistable switch-like, gene-gene interactions in cancer progression ( Shiraishi *et al*., 2010).

## Conclusions

A huge set of data from systems biology suggests that changes occurring within living systems are the result of the activity of multistable switches depending on positive functional loops. The theory of systems and control provides a description of loop system dynamics in terms of a restricted set of mathematical rules, providing a basis for bypassing the drawbacks caused by the biodiversity of life constituents, spanning from molecules to individuals. Such a new conceptualization, focused on interactions rather than on objects, results in a recodification of life in terms of biouniformity, instead of biodiversity, allowing the application of unifying formal analyses on a ground where this approach seemed almost impossible. Accordingly, provided that pathogenic processes affecting healthy individuals must themselves be the result of functional changes, a general theory of pathogenesis can be formulated. Based on this theory, the enormous diversity of pathologies that have been described in the human body can be essentially reduced to a single, unifying pathogenic model, of which any disease would represent a variant. Consequently, each disease can be approached by a standard procedure of analysis aimed at identifying positive loops and their bifurcation parameters, thus addressing critical pharmacological targets. Such a theoretical framework could have a huge impact on pharmacology, medicinal chemistry, and clinical practice. Therefore, given the impasse of many approaches to the study of pathogenesis, this theory would deserve to be validated on specific cases by preclinical and clinical studies, in order to explore the extent to which it can be generalized and represent a turning point in biomedical and pathophysiological research.

## Data availability

No data are associated with this article.
