# Supplementary material for: A general hypothesis of multistable systems in pathophysiology
Source: F1000Res. 2022 Sep 30;11:906. Originally published 2022 Aug 5. [Version 3] doi: 10.12688/f1000research.123183.3 (PMC9530619; doi:10.12688/f1000research.123183.3)
Supplement: Peer review history

# Peer review: Reviewer response for version 3

**DOI:** 10.5256/f1000research.139059.r152154

**Published:** 2022-10-03

**Authors:** Sudin Bhattacharya

**Affiliations:** 1 Department of Biomedical Engineering, Center for Research on Ingredient Safety, Michigan State University, East Lansing, MI, USA

The author has responded to my comments in a satisfactory manner. Thanks for considering my suggestions.

Is the topic of the opinion article discussed accurately in the context of the current literature?

Partly

Are arguments sufficiently supported by evidence from the published literature?

Partly

Are all factual statements correct and adequately supported by citations?

Yes

Are the conclusions drawn balanced and justified on the basis of the presented arguments?

Partly

Reviewer Expertise:

Computational biology, Bioinformatics, Computational toxicology.

I confirm that I have read this submission and believe that I have an appropriate level of expertise to confirm that it is of an acceptable scientific standard.

# Peer review: Reviewer response for version 2

**DOI:** 10.5256/f1000research.137760.r149508

**Published:** 2022-09-22

**Authors:** Sudin Bhattacharya

**Affiliations:** 1 Department of Biomedical Engineering, Center for Research on Ingredient Safety, Michigan State University, East Lansing, MI, USA

The author offers an interesting hypothesis: that multistability, specifically bistability, underlies transitions from health to disease. The basis and implications of this hypothesis are discussed, and a quantitative analysis of diseases is proposed by identifying underlying molecular feedback loops and bifurcation parameters, which can then be used as potential therapeutic targets.

 However, while switching among multiple stable steady states does indeed underlie many physiological processes like the cell cycle, it is not obvious that all disease progression is characterized by abrupt switch-like transitions from healthy to diseased states. For example, development of diabetes, heart disease, or obesity is presumably a gradual process. This is a distinction that the author may want to address. Further, even for diseases that are indeed marked by multistability, identification of all underlying molecular feedback loops and the bifurcation parameters is likely to be a daunting task.

 Another issue worth noting is that the phenomenon of bistability arising from molecular feedback loops concerns behavior at the level of individual cells, not necessarily for whole tissues or organisms. Even among cells of the same type, noisy gene expression can obscure discrete switching and make the transition from one state to another more gradual at the aggregate level. Multi-omic single-cell studies may help clarify whether there are indeed underlying switches or bifurcations behind development of different diseases. It would be helpful to address this point in the manuscript.

 As a final point, I would suggest that the author include some discussion of the theory of critical state transitions as proposed by Scheffer et al ^1^, which suggests that switch-like transitions in natural phenomena may be predicted by early warning signals like increased fluctuation of system variables. This idea has been explored in the context of disease progression ^2^.

 Overall, this manuscript provides an interesting and potentially useful hypothesis about disease development.

Is the topic of the opinion article discussed accurately in the context of the current literature?

Partly

Are arguments sufficiently supported by evidence from the published literature?

Partly

Are all factual statements correct and adequately supported by citations?

Yes

Are the conclusions drawn balanced and justified on the basis of the presented arguments?

Partly

Reviewer Expertise:

Computational biology, Bioinformatics, Computational toxicology.

I confirm that I have read this submission and believe that I have an appropriate level of expertise to confirm that it is of an acceptable scientific standard, however I have significant reservations, as outlined above.

## References

[1] : Early-warning signals for critical transitions. *Nature* .2009;461(7260) : 10.1038/nature08227 53-9 10.1038/nature08227 19727193

[2] : Identifying critical transitions in complex diseases. *Journal of Biosciences* .2022;47(2) : 10.1007/s12038-022-00258-7 10.1007/s12038-022-00258-7 36210727PMC9018973

# Response

**Published:** 2022-09-24

**Authors:** Bruno Burlando

**Affiliations:** University of Genoa, Italy

**I wish to thank the reviewer for appreciating the article and providing various useful suggestions. I have now realized and submitted a new version. Responses to the reviewer's comments and main changes in the new version follow (author in bold):**

 Rev. However, while switching among multiple stable steady states does indeed underlie many physiological processes like the cell cycle, it is not obvious that all disease progression is characterized by abrupt switch-like transitions from healthy to diseased states. For example, development of diabetes, heart disease, or obesity is presumably a gradual process. This is a distinction that the author may want to address.

**Au. This point deserved to be discussed and has been addressed in the paragraph entitled “A loopomic hypothesis in pathophysiology”. Specifically, it has been clarified that the development of diseases observed in clinical setting, appearing as gradual progressions rather than abrupt changes, is the result of cascade events realizing complex patterns that hinder disease management. Conversely, my hypothesis assumes the occurrence of, and is focused on, multistable transitions as point-like events at the root of these processes.**

  Rev. Further, even for diseases that are indeed marked by multistability, identification of all underlying molecular feedback loops and the bifurcation parameters is likely to be a daunting task

**Au. This point had already been addressed after previous revision. But now, also due to other changes in this revision, I have subdivided the former paragraph “A loopomic hypothesis in pathophysiology” into three ones, by introducing two new paragraphs “Limitations to the hypothesis” and “Hypothesis validation”.**

 Rev. Another issue worth noting is that the phenomenon of bistability arising from molecular feedback loops concerns behavior at the level of individual cells, not necessarily for whole tissues or organisms. Even among cells of the same type, noisy gene expression can obscure discrete switching and make the transition from one state to another more gradual at the aggregate level. Multi-omic single-cell studies may help clarify whether there are indeed underlying switches or bifurcations behind development of different diseases. It would be helpful to address this point in the manuscript.

**Au. This point has been addressed in the new paragraph “Limitations to the hypothesis” by closely following the reviewer indications.**

 Rev. As a final point, I would suggest that the author include some discussion of the theory of critical state transitions as proposed by Scheffer et al1, which suggests that switch-like transitions in natural phenomena may be predicted by early warning signals like increased fluctuation of system variables. This idea has been explored in the context of disease progression2.

**Au. This point has been addressed in the new paragraph “Hypothesis validation” by highlighting the importance of multistable transition analysis also in preventive healthcare survey.**

**A total of five additional references have been quoted in the new version of the manuscript and added to the list at the end of text. The complex of changes has considerably improved the contents of the article.**

# Peer review: Reviewer response for version 2

**DOI:** 10.5256/f1000research.137760.r148428

**Published:** 2022-09-01

**Authors:** Rodrigo A. Mora-Rodríguez

**Affiliations:** 1 Research Center on Surgery and Cancer (CICICA), Campus Rodrigo Facio, University of Costa Rica, San José, Costa Rica

The author has addresses all my concerns. Congratulations on this beautiful review.

Is the topic of the opinion article discussed accurately in the context of the current literature?

Partly

Are arguments sufficiently supported by evidence from the published literature?

Yes

Are all factual statements correct and adequately supported by citations?

Yes

Are the conclusions drawn balanced and justified on the basis of the presented arguments?

Yes

Reviewer Expertise:

Systems Biology, miRNA research, cancer, virology

I confirm that I have read this submission and believe that I have an appropriate level of expertise to confirm that it is of an acceptable scientific standard.

# Peer review: Reviewer response for version 1

**DOI:** 10.5256/f1000research.135266.r146862

**Published:** 2022-08-18

**Authors:** Rodrigo A. Mora-Rodríguez

**Affiliations:** 1 Research Center on Surgery and Cancer (CICICA), Campus Rodrigo Facio, University of Costa Rica, San José, Costa Rica

This is a beautiful opinion article accurately summarizing what we, many systems biologists think and see biological complexity.

 Complexity implies that almost everything is connected within an interaction network and the influence of a perturbation could impact other parts of that network. This network could offer robustness but also, beyond certain level of perturbation lead to a different steady state. 

 Although very interesting, I think that the problem is the word theory. To speak about a general theory, more systematic (and directed) validation is required. I would suggest to change that word to "hypothesis" in the title and along the text.

 Apart from that, this hypothesis is fantastic and I would just like to see something more about how the author suggest that bistability can be identified, specially in complex loops since this is one the main limitations of the study of bistability. Perhaps the work of Angeli et al could be of some interest for the author. ^1^

Is the topic of the opinion article discussed accurately in the context of the current literature?

Partly

Are arguments sufficiently supported by evidence from the published literature?

Yes

Are all factual statements correct and adequately supported by citations?

Yes

Are the conclusions drawn balanced and justified on the basis of the presented arguments?

Yes

Reviewer Expertise:

Systems Biology, miRNA research, cancer, virology

I confirm that I have read this submission and believe that I have an appropriate level of expertise to confirm that it is of an acceptable scientific standard, however I have significant reservations, as outlined above.

## References

[1] : Detection of multistability, bifurcations, and hysteresis in a large class of biological positive-feedback systems. *Proc Natl Acad Sci U S A* .2004;101(7) : 10.1073/pnas.0308265100 1822-7 10.1073/pnas.0308265100 14766974PMC357011

# Response

**Published:** 2022-08-21

**Authors:** Bruno Burlando

**Affiliations:** University of Genoa, Italy

I wish to thank the reviewer for the appreciation of my article. Based on the peer review comments, I have prepared a new version of the article. Responses to the reviewer's comments and main changes in the new version follow (author in bold):

 Rev. Complexity implies that almost everything is connected within an interaction network and the influence of a perturbation could impact other parts of that network. This network could offer robustness but also, beyond certain level of perturbation lead to a different steady state. 

**Author. In the new version I have more accurately addressed the problem of complexity in the interactions among biological functional agents. Specifically, input/output monotonicity has been addressed, being a distinctive feature of these interactions and a key element for the possibility of realizing mathematical models able to provide information for operational interventions on these systems, e.g. resolving a pathological condition.**  

 Rev. Although very interesting, I think that the problem is the word theory. To speak about a general theory, more systematic (and directed) validation is required. I would suggest to change that word to "hypothesis" in the title and along the text.

**Author. The word "theory", when referred to the present proposal, has been substituted with "hypothesis". By this way, also the title has been modified.**

 Rev. Apart from that, this hypothesis is fantastic and I would just like to see something more about how the author suggest that bistability can be identified, specially in complex loops since this is one the main limitations of the study of bistability. Perhaps the work of Angeli et al could be of some interest for the author.

**Author. Together with complexity, the issue of bistability has also been addressed and it has been explained more clearly how it is linked to the identification of bifurcation parameters. These latter are a crucial element of the proposed hypothesis, as being considered main sites of  "engineering" interventions on biological systems, obviously including pathological conditions. The suggested reference has been quoted, together with a few others.**
